# Single and Combined Effects of Meropenem, Valproic Acid, and Ketoprofen on Adult Zebrafish Behavior, Oxidative Stress, and Acetylcholinesterase Activity

**DOI:** 10.3390/ph18081096

**Published:** 2025-07-24

**Authors:** Ionut-Alexandru Chelaru, Roxana Strungaru-Jijie, Mircea Nicoara, Diana Mirila, Alin Ciobica, Dorel Ureche

**Affiliations:** 1 Doctoral School of Geosciences, Faculty of Geography and Geology, “Alexandru Ioan Cuza” University of Iasi, Bd. Carol I, 700505 Iasi, Romania; chelaru.alexandru@yahoo.com; 2 Department of Biology, Faculty of Sciences, “Vasile Alecsandri” University of Bacau, Marasesti Street, 600115 Bacau, Romania; dureche@ub.ro; 3 Research Center Advanced Materials and Technologies (RAMTECH), Department of Exact and Natural Sciences, Institute of Interdisciplinary Research, “Alexandru Ioan Cuza” University of Iasi, Bd. Carol I, 700505 Iasi, Romania; roxana.jijie@uaic.ro; 4 Department of Biology, Faculty of Biology, “Alexandru Ioan Cuza” University of Iasi, Bd. Carol I, 700505 Iasi, Romania; alin.ciobica@uaic.ro; 5 Department of Environmental Engineering, Mechanical Engineering and Agritourism, Faculty of Engineering, “Vasile Alecsandri” University of Bacau, Marasesti Street, 600115 Bacau, Romania; miriladiana@ub.ro; 6 “Ioan Haulica” Institute, Apollonia University, Pacurari Street 11, 700511 Iasi, Romania; 7 “Olga Necrasov” Center, Department of Biomedical Research, Romanian Academy, 010071 Iasi, Romania; 8 Multidisciplinary Medical Research and Development Platform in the North-East Region (CENEMED), “Grigore T. Popa” University of Medicine and Pharmacy of Iasi, 700115 Iasi, Romania

**Keywords:** pharmaceuticals, zebrafish, behavior, oxidative stress, joint effects

## Abstract

**Background**: Pharmaceutical compounds frequently co-occur in environmental waters, but studies on their combined effects on animals and humans remain limited. The present study investigated the individual and combined short-term effects of ketoprofen (Kp, a nonsteroidal anti-inflammatory drug inhibiting cyclooxygenase-2), valproic acid (VPA, an anticonvulsant acting as a voltage-gated sodium channel modulator), and meropenem (Mp, a β-lactam antibiotic) at environmentally relevant concentrations on zebrafish behavior, acetylcholinesterase (AChE) activity, and oxidative status. **Methods**: Adult zebrafish were exposed for 4 days to Kp, VPA, Mp, and their binary and ternary mixtures. Behavioral effects were assessed using 3D novel tank and social behavior tests, while the oxidative stress response was assessed through malondialdehyde (MDA) content, superoxide dismutase (SOD), and glutathione peroxidase (GPx) activities. **Results**: Zebrafish exposed to Mp showed a notable increase in immobility, whereas those exposed to VPA and Mp + Kp exhibited a significant augmentation of average velocity and counter-clockwise rotations. All treated groups exhibited a notable increase in the time spent near the walls (thigmotaxis), and except for the control and Mp-exposed zebrafish, the other groups mostly stayed in the bottom tank zone (geotaxis). Kp, VPA + Kp, and VPA + Mp + Kp treatments impaired social behavior, with zebrafish displaying less interest in conspecifics. Biochemical analysis demonstrated that both the individual drugs and their combination caused oxidative stress, characterized by decreased GPx activity and increased SOD activity and MDA levels. Moreover, AChE activity was more strongly inhibited in zebrafish exposed to the binary and ternary mixtures than to individual drugs. **Conclusions**: The results indicate that acute exposure to individual and/or combined pharmaceuticals induces behavioral changes, oxidative damage, and AChE inhibition in zebrafish, highlighting the need to assess the effects of pharmaceutical mixtures for comprehensive ecosystem risks evaluation.

## 1. Introduction

Annually, thousands of tons of pharmaceuticals are used to treat, prevent, or diagnose diseases [1]. Since they cannot be completely degrade by conventional wastewater treatment plants (WWTPs), the drugs and their transformation products reach aquatic environments [2]. Therefore, pharmaceutical pollutants, such as antimicrobial and antidiabetic agents, antidepressants, anticonvulsants, analgesics, and nonsteroidal anti-inflammatory drugs, have gained significant attention all around the world due to their widespread distribution, persistence in the environment for extended periods, and harmful effects on aquatic and terrestrial ecosystems. For instance, Kp, a nonsteroidal anti-inflammatory drug used to treat pain, fever, inflammation, and swelling, has been detected at a level of 10 μg/L in Costa Rican surface waters [3], 9.3 μg/L in the Elbe basin in Czech Republic [4], 10.7 ± 7.3 μg/L in raw sewage from Poland [5], and between 3.6 and 10 μg/L in raw hospital wastewater [6], while the maximum concentration of Kp measured in water of the eastern part of the Gulf of Finland was 4.45 μg/L [7]. In addition, the Kp mean concentrations were found in the range of 0.30–1.36 μg/L and 0.21–0.41 μg/L in the influents and effluents WWTPs from Seville (Spain) [7], respectively, which were lower than those reported for WWTPs from southwestern India (influents: 3–41 μg/L and effluents: 0.6–8 μg/L) [8]. Moreover, only a few studies had been performed to study the effects induced by Kp on aquatic organisms, assessing its effects on zebrafish embryo development, antioxidant enzyme activity, lipid peroxidation level, behavior, and the liver morphology of adult zebrafish [2,9,10]. Rangasamy and co-workers’ results demonstrated that during 42 days of exposure to different levels of Kp (1, 10, and 100 μg/L), the activity of several antioxidant enzymes (e.g., SOD, CAT, GSH, and GPx) decreased, whereas an increase in glutamic oxaloacetic transaminases (ASTs), glutamic pyruvic transaminases (ALTs), and lactate dehydrogenase (LDH) activities was noticed [9]. Moreover, behavioral changes were observed in adult zebrafish exposed to Kp, the fish’s swimming speed increasing immediately after Kp administration [9]. Also, histopathological injuries occurred in the liver of zebrafish exposed to 100 μg/L Kp [9]. On the other hand, the exposure of zebrafish embryos to Kp induced hatching inhibition and decreased/increased heart rates and morphological alterations (e.g., pericardial edema, yolk sac edema, spinal curvature, and lack of pigmentation) [9,10]. The acute experiments on zebrafish revealed EC50 and LC50 values for Kp of 1.91 and 1.52 mg/L, respectively [10]. In addition, the Kp treatment induced alterations in the swimming activity and physiological (e.g., mandible movement, thoracic limb activity, and heart rate) and biochemical (e.g., CAT, GSTs, and COX) parameters of *Daphnia magna* [1,11]. Also, the results showed negative effects for this compound on the growth and development of *Cyprinus carpio* embryos and larvae [12].

Furthermore, Mp, a carbapenem beta-lactam antibiotic with superior activity against anaerobic microorganisms, Gram-positive and Gram-negative pathogens [13], was found in surface waters, with concentrations ranging from 169 ng/L to 1.07 μg/L [14,15,16]. In Singapore hospital wastewater samples, the highest recorded level of Mp reached 1.07 μg/L [14]. Moreover, Mp was detected in 71% of the water samples collected along the Zenne River, which had the highest value of 330 ng/L, and in 27% of sewage water samples from Brussels, with a maximum concentration of 0.95 μg/L [15]. In contrast, the average concentrations of Mp in the influent and effluent of WWTPs from Zagreb (Croatia) were estimated at 169 ± 261.2 ng/L and 260.2 ± 271 ng/L, respectively [16]. Despite the studies on bacteria-injected zebrafish embryos treated with Mp [17] and the mechanism of Mp-induced kidney injury [18], the impact of Mp on zebrafish behavior has not yet been explored in zebrafish. Thus, Habjan et al. showed the 100% survival of *S. pneumoniae*-infected embryos after treatment with Mp at concentrations of 1x or 10x the minimal inhibitory concentration (MIC) [17]. Previous research has found that exposure of adult zebrafish to Mp for 96 h induced nephrotoxicity [18]. Additionally, Guzman-Tordecilla et al. [19] observed that *Lemna minor* exposed to various Mp treatments exhibited physiological alterations and oxidative stress, evidenced by significant augmentation of the SOD, CAT, and APX activity.

VPA is a broad-spectrum antiseizure drug whose fetal exposure is associated with cognitive and neurological impairments, including neural tube malformations, hyperactivity, and an increased risk of developing autism spectrum disorder (ASD) [20,21]. A concentration of 130 ng/L VPA was found in the influent samples from the Back River WWTP in Baltimore (United States) [22], while levels reached up to 2.6 μg/L in Lake Mälaren (Sweden) [23]. Additionally, VPA was detected in five WWTPs in Santorini, with influent concentrations ranging from 366.4 ng/L in the Emporio region to 3008 ng/L in the Fira region [24]. Most research papers have focused on the neuroanatomical and behavioral changes associated with ASD induced by VPA embryonic exposure, such as zebrafish, rather than investigating its effects using environmentally relevant concentrations. Consequently, embryos were subjected to varying doses of VPA for different durations and observed during different life stages [20,25,26]. For example, 5 h post-fertilization (hpf) zebrafish embryos were treated with 140 μg/L for 24 and 48 h and analyzed at 3 and 4 weeks post-fertilization [20], while in another study, 4 hpf embryos were exposed to 6.9 g/L of VPA for 48 h, and the effects were studied during the larval and adult stages [26]. Additionally, zebrafish embryos were exposed to 6.9 mg/L VPA during the first 48 hpf and assessed at 6, 70, and 120 days post-fertilization (dpf) [25].

Limited research exists on the toxicity of exposure to pharmaceutical mixtures, although these compounds tend to occur in mixtures in aquatic environments [27]. According to previous research, the simultaneous presence of multiple chemicals in the environment could exhibit a higher or lower toxicity relative to a single agent [28,29,30,31]. The co-exposure to chlorothalonil and prochloraz decreased the impact on metabolism in zebrafish embryos and larvae [28]. A study conducted by our group validated the antagonistic effects on adult zebrafish behavior and oxidative stress induced by short-term exposure to a mixture of cadmium, nickel, and deltamethrin [29]. In addition, the exposure of zebrafish larvae to binary, ternary, quaternary, and quinquenary mixtures of pesticides for 96 h exhibited synergistic effects [30]. Similarly, another study found more pronounced behavioral effects for zebrafish larvae treated simultaneously with carbendazim and fipronil, compared to those exposed to pesticides individually [31]. Likewise, polyethylene or polypropylene in combination with VPA resulted in significant biochemical and behavioral effects in adult zebrafish [32]. Moreover, simultaneous treatment with Cd and Kp for 42 days resulted in biochemical, behavioral, cognitive, and histopathological changes in adult zebrafish [33], whereas parental exposure to Kp in the presence of Cd had a greater effect on offspring (F1) development, modifying heart function and locomotor activity [34]. Synergistic effects were reported for ternary combinations of iprodione and pyraclostrobin combined with pyrimethanil or acetamiprid, while an antagonistic effect was observed from the quaternary mixture of iprodione, pyrimethanil, pyraclostrobin, and acetamiprid [35].

Zebrafish (*Danio rerio*) are a sensitive and widely used alternative model organism for assessing the (neuro)toxicity of hazardous chemicals due to their low cost; high fecundity; rich behavioral responses; and functional, molecular, and genetic similarity to mammals [36,37]. Their conserved neurotransmitter systems and close genetic resemblance to humans also make them a valuable model in neurological research [38]. In particular, the behavioral studies performed on early-stage (post-hatched larvae) and adult zebrafish have provided new insights into the sublethal effects of various contaminants [39,40]. Common behavioral assays using zebrafish adults include the novel tank test and light/dark test for anxiety-like behavior; T-maze test for social, aggressive, memory, and learning behavior; and open field test for locomotion and exploratory activity [41,42]. These assays are sensitive because alterations in zebrafish behavioral traits occur even at very low pollutant concentrations and after short-term exposure [29,43,44]. Furthermore, the complex fish behavioral responses associated with reproduction, predator avoidance, foraging, and social interaction provide valuable information about the possible health and environmental effects of contaminants [45,46,47]. Moreover, biochemical analyses are frequently employed alongside behavioral assays to add valuable insight into the possible mechanisms underlying observed behavioral changes [48,49,50,51].

The present research evaluated the toxicity of three pharmaceutical compounds on adult zebrafish (*Danio rerio*) through oxidative stress markers, behavioral assays, and AChE activity. The effects of Kp, Mp, and VPA were assessed individually and in binary and ternary mixtures over a 96 h exposure period. Behavior assessments were conducted to identify potential neurotoxic alterations, while biochemical analyses provided insights into the cellular mechanisms involved, thereby improving comprehension of the ecotoxicological risks associated with the drugs used in medicine. To the best of our knowledge, no information is currently available regarding the toxicity of the combined mixture of these pharmaceuticals in aquatic organisms.

## 2. Results

### 2.1. Behavior Analysis

The 3D novel tank and social behavior tests were conducted to evaluate the possible effects of medicines on locomotor activity, exploratory behavior, and social interaction.

#### 2.1.1. Novel Tank Test

**Figure 1:**
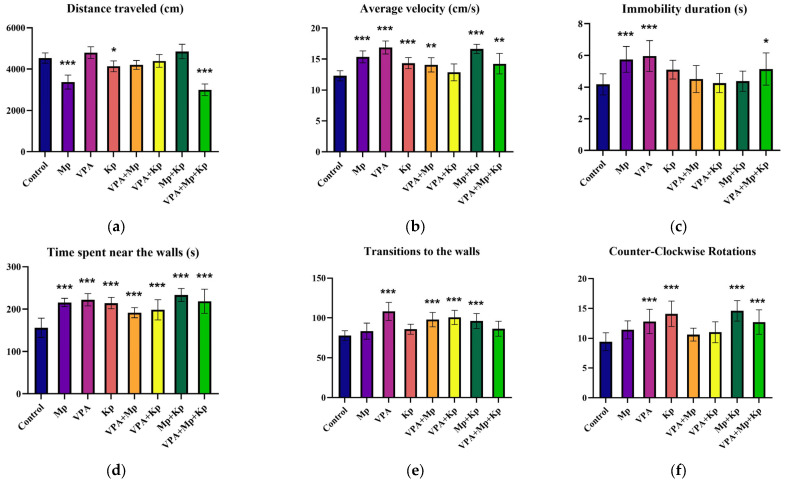
Behavioral changes in adult zebrafish treated with single and combined pharmaceuticals (Mp = 1 µg/L; VPA = 3 µg/L; Kp = 5 µg/L) for 96 h. Behavioral parameters measured include (**a**) total distance traveled (cm), (**b**) mean speed (cm/s), (**c**) immobility duration (s), (**d**) time spent near the walls (s), (**e**) frequency of transitions to the walls, and (**f**) counter-clockwise rotations. Data are presented as mean ± SD (*n* = 10 animals per group, with a 1:1 male-to-female sex ratio) and were analyzed using one-way ANOVA followed by Dunnett’s test. Statistically significant differences are indicated as follows: * *p* < 0.05, ** *p* < 0.01, and *** *p* < 0.001.

**Figure 2:**
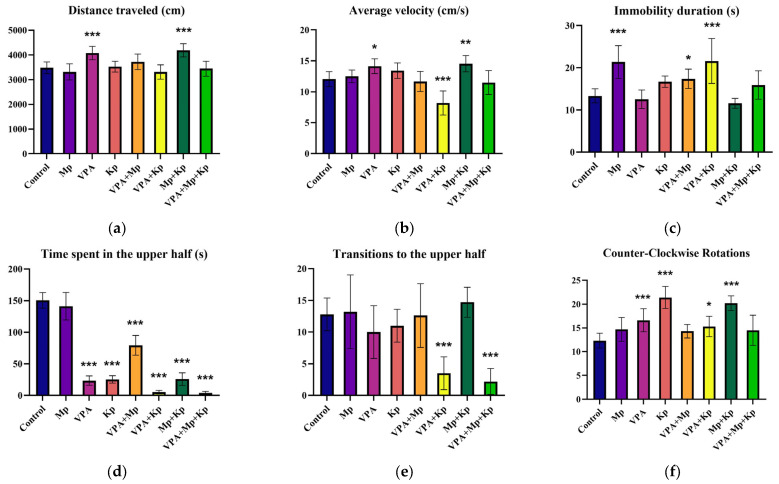
Behavioral changes in adult zebrafish treated with single and combined pharmaceuticals (Mp = 1 µg/L; VPA = 3 µg/L; Kp = 5 µg/L) for 96 h. Behavioral parameters measured include (**a**) total distance traveled (cm), (**b**) mean speed (cm/s), (**c**) immobility duration (s), (**d**) time spent in the upper half (s), (**e**) transitions to the upper half, and (**f**) counter-clockwise rotations. Data are presented as mean ± SD (*n* = 10 animals per group, with a 1:1 male-to-female sex ratio) and were analyzed using one-way ANOVA followed by Dunnett’s test. Statistically significant differences are indicated as follows: * *p* < 0.05, ** *p* < 0.01, and *** *p* < 0.001.

As shown in Figure 1 and Figure 2, the individual and combined short-term exposure to Kp, VPA, and Mp significantly altered the swimming behavior of adult zebrafish compared to the control group. For instance, the Mp and VPA + Mp + Kp groups showed a significant decrease (*p* < 0.001) in the total distance traveled compared to the control, as well as fish exposed to single compounds and binary mixtures (Figure 1a), during the 5 min of video recording with a camera placed above the test tank. Interestingly, the treatment with VPA alone for 96 h induced the highest increase in mean velocity, showing a 1.37-fold rise relative to the control (*p* < 0.001), while in the mixture with Mp (*p* = 0.004), it increased by 1.14-fold, with Kp (not significant) by 1.05-fold, and with Mp + Kp (*p* < 0.001) by 1.16-fold (Figure 1b). Conversely, the average swimming speed is higher for the Mp + Kp binary mixture (16.63 ± 0.76 cm/s) than for fish exposed to the Kp alone (14.34 ± 0.88 cm/s). Notably, exposure to VPA (*p* < 0.001 vs. control) and Mp (*p* < 0.001 vs. control) led to an approximately 1.4-fold increase in the immobility duration (Figure 1c). On the other hand, acute exposure of zebrafish to pharmaceuticals significantly increased the time zebrafish spent near the walls, a behavior known as thigmotaxis (Figure 1d). For example, the total time spent near the walls was augmented by 1.5-fold (*p* < 0.001) in the Mp + Kp-exposed group. Supportively, the Mp, VPA, VPA + Mp, VPA + Kp, and Mp + Kp groups (*p* < 0.001) made more transitions to the walls of the tank than the control group (Figure 1e). Furthermore, after 96 h exposure to VPA, Kp, Mp + Kp, and VPA + Mp + Kp, a significant increase in the number of counter-clockwise rotations (*p* < 0.001 vs. control) was observed (Figure 1f).

On the contrary, zebrafish exposed to VPA and MP + Kp revealed a considerable rise in the distance traveled (*p* < 0.001), compared to the control group, during the 5 min of video recording with a camera placed in front of the test tank (Figure 2a). Moreover, the VPA + Kp group showed a significant decrease in mean velocity (8.17 ± 1.96 cm/s), while the VPA (14.13 ± 1.19 cm/s) and Mp + Kp (14.53 ± 1.31 cm/s) groups had the highest average speed, in contrast with the control group (12.05 ± 1.21 cm/s) (Figure 2b). As seen in Figure 2c, fish treated with Mp and VPA + Kp for 4 days presented a significant increase (*p* < 0.001) in immobility duration by approximately 1.6-fold. Unlike the control and Mp-exposed zebrafish that explored the entire aquarium, the fish exposed to the other single and combined pharmaceutical treatments explored mostly the bottom part of the tank (geotaxis), suggesting anxiety-related behavior (Figure 2d). Although zebrafish treated with VPA and Kp alone and in combination with Mp frequently entered the upper half, the overall duration spent in the upper part of the tank was significantly decreased in comparison to the control group (Figure 2e). The frequency of transitions to the upper part of the aquarium was significantly lower (*p* < 0.001) for the VPA + Kp (3.7-fold) and VPA + Mp + Kp (5.8-fold) groups, in contrast with the control group. Additionally, acute exposure to VPA (*p* = 0.001 vs. control) and Kp alone (*p* < 0.001 vs. control) and in combination with Mp (*p* < 0.001 vs. control) revealed a significant rise in counter-clockwise rotations (Figure 2f).

#### 2.1.2. Social Behavior Test

**Figure 3:**
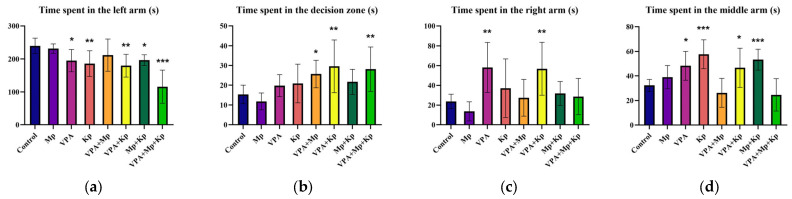
Effects of single and combined pharmaceuticals (Mp = 1 µg/L; VPA = 3 µg/L; Kp = 5 µg/L) acute treatments on adult zebrafish social interaction. Behavioral parameters measured include (**a**) amount of time that zebrafish spent in the left arm, (**b**) decision zone, (**c**) right arm, and (**d**) middle arm. Data are presented as mean ± SD (*n* = 10 animals per group, with a 1:1 male-to-female sex ratio) and were analyzed using one-way ANOVA followed by Dunnett’s test. Statistically significant differences are indicated as follows: * *p* < 0.05, ** *p* < 0.01, and *** *p* < 0.001.

Further, social behavior was assessed by analyzing how individual zebrafish interacted with conspecifics. This test demonstrated that the exposure to VPA, Kp, VPA + Kp, Mp + Kp, and VPA + Mp + Kp at environmentally relevant concentrations for 96 h induced a social interaction deficit in zebrafish. As shown in Figure 3a, the time spent by animals in the left arm, close to the conspecifics, was significantly reduced for Kp alone (*p* = 0.005), in combination with VPA (*p* = 0.001), and ternary mixture-exposed groups (*p* < 0.001), compared to the control group. In addition, short-term exposure to VPA + Mp (*p* = 0.04), VPA + Kp (*p* = 0.002), and VPA + Mp + Kp (*p* = 0.006) led to a significant increase in the time spent in the decision zone (Figure 3b). Moreover, the fish exposed to VPA alone (*p* = 0.002) and in mixture with Kp (*p* = 0.003) spent more time in the right arm, compared with the control animals (Figure 3c). Interestingly, the VPA (*p* = 0.01), Kp (*p* < 0.001), VPA + Kp (*p* = 0.03), and Mp + Kp (*p* < 0.001) groups showed a marked increase in the time spent by fish in the central segment (Figure 3d).

### 2.2. Oxidative Stress Status and Acetylcholinesterase Activity

**Figure 4:**
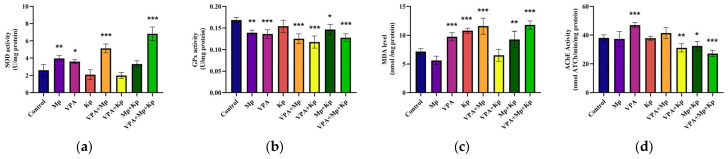
Biochemical parameters of adult zebrafish after 96 h of treatment with single and combined pharmaceuticals (Mp = 1 µg/L; VPA = 3 µg/L; Kp = 5 µg/L). The following biomarkers were assessed: (**a**) SOD (U/mg of protein), (**b**) GPx (U/mg of protein), (**c**) MDA (nmol/mg of protein), and (**d**) AChE (nmol ATCh/min/mg of protein). Data are presented as mean ± SD (*n* = 5) and were analyzed using one-way ANOVA followed by Dunnett’s test. Statistically significant differences are indicated as follows: * *p* < 0.05, ** *p* < 0.01, and *** *p* < 0.001.

The oxidative stress response in zebrafish exposed to Mp, VPA, and Kp individually and in combination was assessed by measuring the activity of two principal antioxidant enzymes (SOD and GPx) and lipid peroxidation levels (MDA). As depicted in Figure 4a–c, individual and combined pharmacological exposure generates variability in the formation of reactive oxygen species (ROS) and its elimination by the antioxidant defense system. According to the results, SOD activity was significantly higher in the Mp (*p* = 0.001), VPA (*p* = 0.02), VPA + MP (*p* < 0.001), and VPA + MP + Kp (*p* < 0.001) treatment groups compared to the control. In contrast, acute exposure to MP (*p* = 0.001), VPA (*p* < 0.001), VPA + Mp (*p* < 0.001), VPA + Kp (*p* < 0.001), Mp + Kp (*p* = 0.02), and VPA + Mp + Kp (*p* < 0.001) significantly decreased GPx antioxidant activity. There was a marked increase in the MDA contents between almost all treated and control groups, except for fish exposed to Mp and VPA + Kp treatments. On the other hand, the AChE activity in zebrafish brains exposed to VPA (*p* < 0.001) increased, while for VPA + Kp (*p* = 0.007), Mp + Kp (*p* = 0.04), and VPA + MP + Kp (*p* < 0.001), it decreased more than that in the controls, as illustrated in Figure 4d.

### 2.3. Pearson’s Correlation Analysis Among Biochemical and Behavioral Parameters

**Figure 5:**
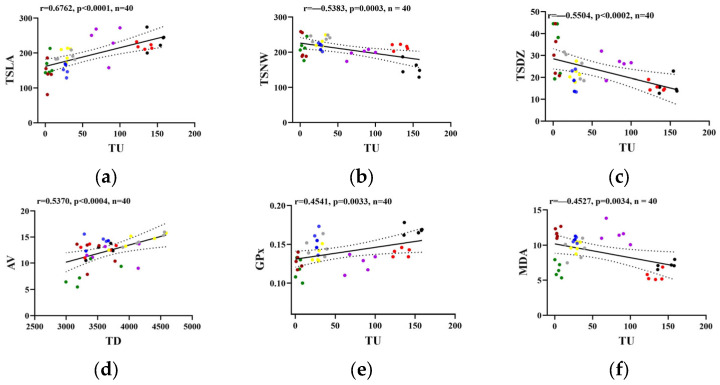

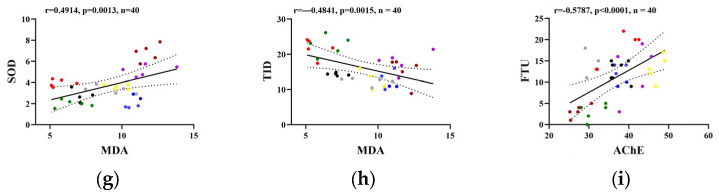
Pearson’s correlation analysis between behavioral and biochemical parameters: (**a**) TSLA and TU, (**b**) TSNW and TU, (**c**) TSDZ and TU, (**d**) AB and TD, (**e**) GPx and TU, (**f**) MDA and TU, (**g**) SOD and MDA, (**h**) TID and MDA, (**i**) FTU and AChE. Data points represent individual zebrafish from the following groups: (●) Control, (●) Mp, (●) VPA, (●) Kp, (●) VPA + Mp, (●) VPA + Kp, (●) Mp + Kp, and (●) VPA + Kp + Mp, exposed to environmentally relevant concentrations for 96 h. The solid line represents the linear regression, while the dashed lines show the 95% confidence interval. Abbreviations: TSLA = time spent in the left arm, TSDZ = time spent in the decision zone, TDS = total distance (side view), AV = average velocity (side view), TID = total immobilization duration (side view), TU = time in upper half, FTU = frequency of transitions to upper half, TSNW = time spent near the walls.

In the current study, the strongest positive correlation was observed between the time spent by zebrafish in the left arm and the time spent in the upper half of the tank (Figure 5a). In contrast, the time spent near the walls and the time in the upper half had the highest negative correlation (Figure 5b). As illustrated in Figure 5a–d, the novel tank swimming parameters were correlated not only among themselves, but also with social interaction variables. Furthermore, mild but significant correlations were found between the GPx activity (r = 0.4541, *p* = 0.0033, *n* = 40) and the MDA level (r = −04527, *p* = 0.0034, *n* = 40) with the time in the upper half (Figure 5e,f). Similarly, DePasquale et al. [52] reported that the time spent by zebrafish in the top portion of the tank was highly associated with other novel tank swimming endpoints, with correlation coefficients ranging between 0.86 for entries to the top and −0.48 for freeze duration. In addition, Popovici et al. revealed a strong positive correlation between the time spent by zebrafish in the light zone (r = 0.6439, *p* < 0.0001, *n* = 135) and in the inner zone (r = 0.6015, *p* < 0.0001, *n* = 135) with the time spent in the top zone [53]. A positive correlation between time spent in the bottom zone and GPx was reported by Heredia-García [50]. Moreover, the MDA level was positively correlated with SOD activity (r = 0.4914, *p* = 0.0013, *n* = 40) but negatively with the total immobility duration (r = −0.4841, *p* = 0.0015, *n* = 40), as shown in Figure 5g,h. In agreement with our results, a previous study evidenced negative correlations between the lipoperoxidation level and several behavioral parameters, such as time spent in the top and freezing duration, while demonstrating a positive association with SOD activity in zebrafish exposed to Bisphenol-A [50]. On the other hand, moderate positive correlations were obtained between the frequency of transitions to the upper half (r = 0.5787, *p* < 0.0001, *n* = 40) and AChE activity (Figure 5i). In line with our results, swimming activity was significantly correlated with AChE activity in crucian carp exposed to sertraline [48]. Gravato et al. revealed a positive association between AChE and covered distance for estuarine fish Pomatoschistus microps exposed for 96 h to copper and mercury [49]. Similarly, positive associations were observed between transitions between top and bottom and total swimming distance with AChE activity in adult zebrafish exposed to Bisphenol-A for 96 h [50].

In summary, the novel tank swimming parameters were associated not only among themselves but also with social interaction variables, AChE activity, and oxidative stress biomarkers, constituting an integrative approach.

## 3. Discussion

According to previous studies, the presence of pharmaceutical chemicals in aquatic environments can affect zebrafish across multiple developmental stages [54]. Furthermore, exposure to drugs induced neurotoxicity in adult zebrafish [55,56], as revealed by both behavioral [57,58,59] and biochemical assays [60]. These drugs were selected based on clinical studies indicating that Mp can act as an antidote in cases of acute VPA poisoning by reducing its plasma levels in humans [61]. Additionally, Kp was included due to its common use in hospital settings. All three pharmaceuticals are frequently detected in environmental waters, which broadens the relevance of the present study [5,6,7,8,14,15,23,24].

In the present study, the first assay performed on the control and treated groups (Mp, VPA, Kp, VPA + Mp, VPA + Kp, Mp + Kp, and VPA + Mp + Kp) was the novel tank test, a standard approach for anxiety-like behavior in zebrafish that assesses exploratory behavior and swimming performance parameters [52,62]. For instance, the Mp and VPA + Mp + Kp treatment groups showed a substantial reduction in the overall distance traveled, while the VPA and Mp + Kp treatment groups exhibited an increase in both the distance traveled and the mean swimming speed, compared to the control group. A similar result was reported by Dhanabal and Durairaj [63], where adult zebrafish treated with VPA through immersion and intraperitoneal injection (100 mg/kg) showed a significant increase in the distance traveled. In addition, pre-administration of chrysin before VPA treatment resulted in a decrease of this locomotor parameter. In contrast, Baronio et al. demonstrated that exposure to VPA (25 μM) during the embryonic stage leads to significant behavioral alterations in adulthood, including reduced distance traveled and decreased noradrenaline, a neurotransmitter involved in attention and cognition [64]. Comparable results were observed in research conducted on zebrafish larvae (7 dpf), where exposure to VPA (500 µM) led to a reduction in the average distance traveled under both light and dark conditions [65]. Similarly, Karimi and co-workers found that both acute and chronic exposure to VPA impair the locomotor activity of zebrafish larvae [66]. Several studies have shown the negative impact of Kp on *Danio rerio,* particularly on embryos, affecting their physiology and potentially causing mortality [9,67]. Moreover, Madesh et al. reported that exposure of adult zebrafish to cadmium (10 μg/L) in the presence of Kp (10 and 100 μg/L) affects cadmium accumulation in the cerebral tissue, underscoring the importance for additional investigation of chemical combinations [33]. Additionally, this test revealed that zebrafish exposed to VPA, Kp, MP + Kp, and a ternary mixture showed a significant increase in counter-clockwise rotations, possibly due to the anxiolytic effects of the pharmaceuticals. According to the literature, increased counter-clockwise behavior [68] indicates higher anxiety levels in zebrafish, and also it serves as a neural marker linked to aggressive behavior [69]. Another important parameter to assess anxiety in organisms is the time spent near walls, known as thigmotaxis or “wall-hugging” [70,71]. It must be noted that all treated groups spent more time near aquarium walls than the control group. Karimi et al. have similarly shown that exposure to VPA led to a significant elevation in the anxiety level associated with thigmotaxis [66]. In contrast with these results, Caioni et al. found that exposure of zebrafish larvae to sodium valproate at concentrations of 10 and 25 mg/L did not significantly affect thigmotaxis and distance traveled. However, extensive anomalies, such as reduced blood flow, pericardial edema, yolk sac deformation, craniofacial deformity, lordosis, and scoliosis, were observed [67].

It is well-established that anxious fish exhibit less exploration in the upper area and tend to remain in the lower zone of the aquarium, often displaying prolonged freezing duration [72]. Currently, limited knowledge exists about Mp effects on behavioral parameters, and no results have been reported for zebrafish to date. For example, exposure to Mp has been shown to cause cognitive deficits, increase exploratory behavior, and alter the expression of signaling molecules in the mouse brain [73]. Herein, the zebrafish treated with Mp exhibited the same behavior as the control group, exploring the upper part of the aquarium. In contrast, all other treated groups spent more time at the bottom of the tank, with a slight improvement observed for the group treated with VPA + Mp, suggesting a reduced exploratory capacity. Also, our results revealed that groups treated with Mp and VPA + Kp exhibited longer freezing periods, providing insight into the state of anxiety. Similarly, modest thigmotaxis and freezing duration along with increased counter-clockwise rotations were observed for zebrafish exposed to lysergic acid diethylamide [74].

Zebrafish exhibit well-defined social behaviors, including a strong affiliative behavior known as shoaling. Their natural tendency is to live in shoals (groups), showing a strong preference for social interaction. Group affiliation is a beneficial survival strategy since it enhances foraging efficiency, supports reproductive behavior, and provides protection against predators [75]. Consequently, studying zebrafish responses to their conspecifics (social behavior) can help in understanding the effects of environmental contaminants. For example, the fish treated with VPA + Kp and VPA + Mp + Kp exhibited social impairments, as evidenced by their preference to swim in the right arm compared to the control group. According to previous studies, VPA can affect some key behavioral endpoints, such as zebrafish sociability. For example, in both larvae [76] and adults [77], treatment with VPA impaired social behavior and increased the signs of hostility and stress. Aside from the altered social interaction, an increase in repetitive behaviors was also observed [52]. In line with our results, altered social behavior has also been reported for other pharmaceuticals, such as sertraline (antidepressant) [78], fentanyl (opioid analgesic) [79], and amoxicillin (antibiotic) [73].

Environmental pollutants may promote oxidative stress in zebrafish by elevating reactive oxygen species (ROS) levels, potentially resulting in cellular damage [80,81,82,83]. Antioxidant enzymes, like SOD, CAT, and GPx, are essential for neutralizing free radicals, thereby mitigating oxidative stress and the preservation of cellular balance. On the other hand, malondialdehyde is a byproduct of lipid peroxidation, frequently used as a biochemical diagnostic of oxidative stress in toxicological studies [84]. Both individually and in combination, drugs can influence the production of free radical species through various mechanisms. On their own, compounds may promote oxidative stress by generating reactive oxygen species (ROS) or by impairing mitochondrial function and antioxidant defenses [85]. When combined, their effects can be additive, synergistic, or antagonistic—potentially amplifying or reducing oxidative damage. For instance, valproic acid undergoes extensive hepatic metabolism to reactive metabolites and has been shown to increase markers of oxidative stress (like malondialdehyde) while decreasing antioxidant enzymes (SOD, CAT, GPx) in rodent and human studies [86]. Similarly, ketoprofen is metabolized in the liver by cytochrome P450 enzymes and correspondingly elevates ROS production [87]. Together, ketoprofen and valproic acid may lead to enhanced oxidative stress through overlapping metabolic pathways and shared depletion of antioxidant defenses, making combined use a potential risk for increased ROS formation. In this study, the presence of VPA in combination with MP and Kp resulted in a significant increase in SOD activity. Moreover, except for the Mp and VPA + Kp groups, all other treated groups showed significant elevation in MDA levels, indicating increased oxidative stress. In addition, GPx activity was also affected by single administration and mixtures, except in the Kp and Mp + Kp treatment groups. Rangasamy et al. [9] revealed that SOD and GPx activities in zebrafish liver tissue were decreased significantly on days 14, 28, and 42, for all studied concentrations (1, 10, and 100 μg/L) compared with the control group. Furthermore, Kp treatments raised MDA levels in a manner that was dependent on both the concentration and time. In addition, Diniz et al. [2] noted a notable rise in the MDA concentration and SOD % inhibition for fish exposed to 1 mg/L ketoprofen over seven days, in comparison with controls. Similarly, a high level of lipid peroxidation occurred after the exposure of zebrafish to Kp and Cd [34]. Consistent with these findings, common carp exposed to chlorpyrifos (80 μg/L) for 14 days revealed that SOD, GST, and MDA activity significantly increased, whereas CAT and GPx activity significantly decreased.

The evaluation of neurotoxicity often involves the measurement of AChE activity, a key biochemical marker frequently used in toxicological studies to evaluate the impact of chemical substances on nervous system functionality [88]. A study by Kim et al. demonstrated that prenatal exposure to VPA led to upregulation of AChE in the prefrontal cortex of rat and mouse offspring. This was also observed in vitro in differentiating cortical neural progenitor cells treated with VPA. The mechanism involved increased acetylation of histone H3 at the AChE promoter, suggesting epigenetic regulation. These findings support the idea that VPA can indirectly modulate cholinergic signaling through AChE upregulation, potentially contributing to neurodevelopmental toxicity [89]. In contrast, there is no direct evidence in the current pharmacological literature that either ketoprofen or meropenem interact with or inhibit AChE. The current investigation demonstrated that AChE activity was affected by exposure to pharmaceuticals; thus, an increase was observed in the VPA-treated group, while a decrease was noted in zebrafish exposed to the VPA + Kp and VPA + Mp + Kp mixtures. According to the literature, both increases and decreases in AChE activity have been associated with cognitive and behavioral impairments [39]. VPA has been reported to upregulate AChE activity [90], potentially as a compensatory response to altered cholinergic signaling [91]. However, when VPA is combined with other pharmaceuticals, such as KP or MP, the resulting mixture may exert additive or synergistic neurotoxic effects, including oxidative stress or disruption of membrane integrity, which could inhibit AChE activity [92]. Studies suggest that oxidative damage can suppress AChE function [93], and thus, the presence of additional compounds may shift the balance from compensation to inhibition. These findings are in accordance with a study conducted by Massei et al.: a water sample collected from the Danube River, containing 276 emerging contaminants from different classes, significantly inhibited zebrafish AChE activity at the end of the 4-day exposure period [94]. Conversely, elevated AChE activity was noted in groups administered paracetamol, carbamazepine, metformin, and trimethoprim at environmentally relevant doses, individually and in combination, during a 7-day exposure duration [95]. Each chemical influences oxidative balance and neuronal functionality through distinct mechanisms. When combined, these compounds may interact synergistically, resulting in increased oxidative stress, impaired antioxidant defenses, and a level of toxicity that exceeds the sum of their individual effects.

In summary, zebrafish are an efficient model for studying the effects of hazardous chemicals, including pharmaceutical compounds, in aquatic organisms. Moreover, the simultaneous coexistence of various compounds in the aquatic environment can led to synergistic, antagonistic, or cumulative toxicological effects, highlighting the need for further studying of chemical mixtures and the incorporation of mixture-based data into the development of regulatory thresholds and standards.

## 4. Materials and Methods

### 4.1. Chemicals and Test Substances

Bradford Reagent (B6916, Quality Level 200), Bovine Serum Albumin (A8022, ≥96%, Quality Level 300), ethanol EMSURE^®^ (159010, 96%, Quality Level 300), acetylthiocholine iodide (ATCh, A5751, ≥98%), 5,5-dithio-2,2-nitrobenzoic acid (DTNB, 322123, ≥98%, Quality Level 100), acetone (179124, ≥99.5%, Quality Level 200), glacial acetic acid (A6283, ≥99%, Quality Level 200), Superoxide Dismutase Assay Kit (SOD, 19160), the Glutathione Peroxidase Cellular Activity Assay Kit (GPx, CGP1), and the Lipid Peroxidation Assay Kit (MDA, MAK085) were purchased from Merk, Darmstadt, Germany. The ketoprofen liquid form (50 mg/mL), meropenem powder (vials of 500 mg), and valproic acid tablets (500 mg) were purchased from local pharmacies. The Kp, Mp, and VPA stock solutions with a concentration of 100 mg/L were prepared daily by dissolving in distilled water.

### 4.2. Zebrafish Maintenance

Adult wild-type zebrafish (*Danio rerio*) (6 months old) of the AB genetic line, with a mean weight of 0.73 ± 0.1 g and a mean length of 38 ± 4 mm, were acquired from a local supplier (Iasi, Romania) and accommodated to laboratory conditions for 14 days in a 92 L aquarium (measuring 60 cm × 30 cm × 45 cm, length × width × height) prior to the assays. Both genders were housed mixed, with a male-to-female sex ratio of approximately 1:1 in all housing tanks. White paper dividers were placed between adjacent housing tanks and around them to mitigate fish interaction and reduce external stress factors. Behavioral testing occurred in a designated testing room, with all assessments completed between 9:00 AM and 5:00 PM. Each experimental fish underwent the behavioral assays, with one fish from each group assessed daily in a randomized sequence according to housing conditions. The fish were fed twice daily with commercially available dry food (e.g., TetraMinTM flakes) at a level of approximately 1% of the body weight per day, and their environmental conditions were maintained as mentioned in previous protocols [29,88,96].

### 4.3. Experimental Design for Acute Toxicity in Adult Zebrafish

Then, for acute toxicity investigation, the zebrafish were randomly divided into eight groups (*n* = 30 organisms per group, with a 1:1 male-to-female sex ratio); acclimated for 3 days in 10 L aquariums (measuring 30 cm × 20 cm × 17 cm, length × width × height); and exposed to water (control), Kp, Mp, and VPA alone or in combination for 96 h, as illustrated in Table 1. The pharmaceutical concentrations were selected based on environmental levels previously reported in the literature [5,6,7,8,14,15,23,24]. During the 4 days, the exposure solutions were changed every 24 h. The 96 h exposure period was selected in accordance with OECD Test No. 203 (Fish, Acute Toxicity Test), which recommends this duration for assessing short-term toxic effects in fish. At the end of the 96 h of exposure, the locomotor activity and social behavior of each fish (*n* =10, with a 1:1 male-to-female sex ratio) from each group were monitored for 5 min, following 30 s of acclimation in the test tank/T-maze system.

### 4.4. Behavioral Analysis

#### 4.4.1. Three-Dimensional Novel Tank Test

**Figure 6:**
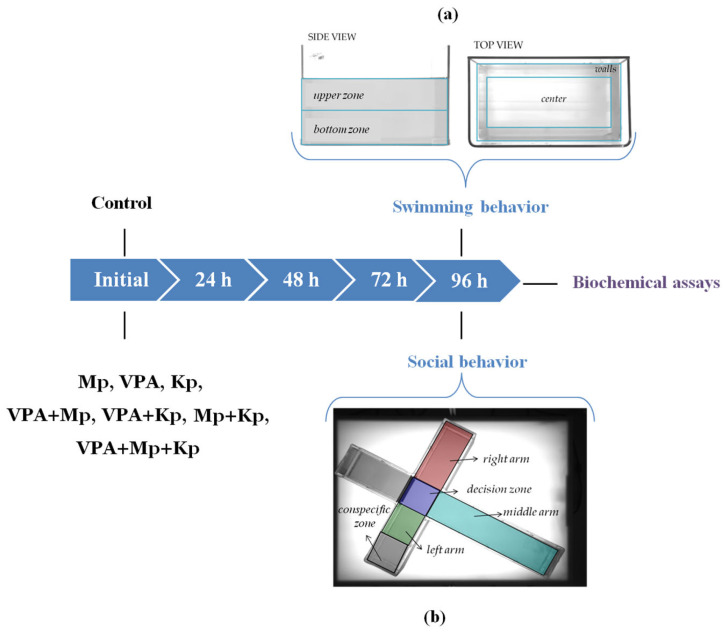
Schematic diagram of the experimental setup. (**a**) 3D novel tank test and (**b**) social behavior test.

A rectangular tank (measuring 30 cm × 20 cm × 17 cm, length × width × height), filled with 6 L of dechlorinated tap water, was used with two cameras positioned orthogonally to one another to obtain a top view and a front view of fish swimming. Moreover, the side and top parts of the tank were divided into two different zones, as shown in Figure 6a. The exploratory behavior of each adult zebrafish was recorded and analyzed with EthoVision XT software, version 16 (Noldus Information Technology, Wageningen, The Netherlands). The following parameters were analyzed: distance traveled (cm), average velocity (cm/s), immobility duration (s), time spent near the walls or in the upper half (s), transitions to the walls or to the upper half, and counter-clockwise rotations.

#### 4.4.2. Social Preference Test

This experiment was performed in a T-shaped maze (measuring 70 cm × 50 cm × 10 cm, length × height × width) containing five regions (left arm, decision zone, right arm, middle arm, and conspecific zone) separated by transparent plastic partitions (Figure 6b). The maze was filled with 5 L of water, and 3 fish were placed in the conspecific zone at the end of the left arm. The period the experimental fish spent in each zone and the frequency of entries into the decision zone and left, right, and middle arms were measured using the same software as the 3D novel tank test.

### 4.5. Biochemical Analysis

Following behavioral analysis, zebrafish were euthanized, employing the hypothermic shock approach (2 °C to 4 °C) in accordance with the AVMA Guidelines [97]. Once the animals showed no vital signs, with the help of a 7 mL KIMBLE Dounce tissue grinder set (Sigma Aldrich, St. Louis, MO, USA), each fish was homogenized in 10% (*w*/*v*) ice-cold 0.1 M phosphate buffer saline (pH 7.4). Following centrifugation at 5500 rpm for 15 min (4 °C), the resulting supernatants were used for biochemical analyses, and proteins were estimated using the Bradford method.

#### 4.5.1. Assessment of Enzymatic Antioxidants and of Lipid Peroxidation

To determine the SOD and GPx activities and the MDA level, we used commercial assay kits (19160, CGP and MAK085 from Merck, Darmstadt, Germany), in accordance with the manufacturers’ guidelines.

#### 4.5.2. Evaluation of Acetylcholinesterase Activity

The AChE activity was assessed using the method described by Boiangiu et al. [98], with slight modification. Initially, 2 fish brains per one independent sample were homogenized in 10 volumes of extraction buffer and centrifuged (14,000 rpm, 10 min, 4 °C). Then, 50 mL of the supernatant was mixed with 450 mL of sodium phosphate buffer solution (pH 7.4, 0.25 M), 50 mL of DTNB (1 mM), and 25 mL ATCh (5 mM). After 10 min of incubation at 37 °C, the reaction was stopped by adding 900 mL of acetone, and the absorbance was measured at 412 nm by using the Specord 210 Plus from Analytik Jena, Germany. The AChE activity was expressed as nanomoles of ATCh hydrolyzed/min/mg protein.

### 4.6. Statistical Analysis

The data normality and distributions were studied by the Shapiro–Wilk test, using the Graph Pad Prism 10.4.1 program (San Diego, CA, USA). Additionally, statistical significance among the groups was obtained by employing the one-way ANOVA test, followed by Dunnett’s test. The results are expressed as the mean ± standard deviation (SD). The level of significance was set to at least *p* < 0.05. We also performed a Pearson correlation analysis to study the relationship between behavioral and biochemical parameters (95% confidence interval). G*Power software, version 3.1.9.7 (University of Düsseldorf, Düsseldorf, Germany) was used to calculate the required sample size for detecting a medium effect size, with a significance level of α = 0.05 and a power of 80%.

## 5. Conclusions

Overall, our findings demonstrate that short-term exposure to environmentally relevant concentrations of individual and combined pharmaceuticals (VPA, Kp, and Mp) alters zebrafish behavior, induces oxidative stress, and inhibits AChE activity. Specifically, exposure to VPA, Kp, Mp + Kp, and VPA + Mp + Kp induced anxiety-related behaviors, including increased counter-clockwise rotations, thigmotaxis, and immobility. VPA, Kp, and VPA + Kp treatments caused the most pronounced impairment in exploration and social interaction, indicating potential neurotoxicity. Notably, the VPA + Mp mixture suggested an antagonistic interaction, whereas the VPA + Kp, Mp + Kp, and VPA + Mp + Kp combinations showed synergistic toxicity, reflected in both behavioral and biochemical alterations. Regarding the biochemical results, the VPA, Kp, VPA + Kp, and VPA + Mp + Kp groups exhibited significant oxidative imbalance, and AChE activity was strongly inhibited in the VPA + Kp and VPA + Mp + Kp groups. Moreover, time spent by zebrafish in the upper half of the tank exhibited the strongest correlations with both behavioral and biochemical parameters. These results provide further insight into the toxic effects induced by the simultaneous presence of various pharmaceuticals in aquatic ecosystems. Taking into account the high molecular and genomic similarities between zebrafish and other vertebrates, including humans, the observed behavioral and biochemical impairments suggest that exposure to pharmaceutical mixtures in water environments may pose significant risks not only to aquatic organisms but potentially to human health. Further research is necessary to elucidate the precise mechanisms underlying the side effects of pharmaceutical compounds on zebrafish. Moreover, evaluating the potential effects of drugs separately in males and females, as well as during the complete life cycle of zebrafish, would significantly enhance our understanding of long-term and sex-specific toxicity.

## Figures and Tables

**Table 1:** Concentrations of the single compounds and binary and ternary mixtures used to measure the behavioral changes and oxidative status.

Compounds	Mp (μg/L)	Kp (μg/L)	VPA (μg/L)
Mp	1	0	0
Kp	0	5	0
VPA	0	0	3
VPA + Mp	1	0	3
VPA + Kp	0	5	3
Mp + Kp	1	5	0
VPA + Mp + Kp	1	5	3

## Data Availability

The original contributions presented in this study are included in the article. Further inquiries can be directed to the corresponding author.

## Abbreviations

The following abbreviations are used in this manuscript:

Mp	Meropenem
Kp	Ketoprofen
VPA	Valproic acid
LD	Linear dichroism
ROS	Reactive oxygen species
SOD	Superoxide dismutase
MDA	Malondialdehyde
GPx	Glutathione peroxidase
AChE	Acetylcholinesterase
GSH	Glutathione
GST	Glutathione S-transferase
TSLA	Time spent in the left arm
TDS	Total distance
AV	Average velocity
TID	Total immobilization duration
CCRS	Counter-clockwise rotations
TU	Time in upper half
FTU	Frequency of transitions to upper half
TSNW	Time spent near the wall
FTW	Frequency of transitions to walls
Cd	Cadmium
hpf	Hours post-fertilization
dpf	Days post-fertilization
WWTPs	Wastewater treatment plants
COX	Cyclooxygenase
AST	Glutamic oxaloacetic transaminase
ALT	Glutamic pyruvic transaminase
LDH	Lactate dehydrogenase
APX	Ascorbate peroxidase
ASD	Autism spectrum disorder
